# Seroprevalence of Major Pasture-Borne Parasitoses (Gastrointestinal Nematodes, Liver Flukes and Lungworms) in German Dairy Cattle Herds, Association with Management Factors and Impact on Production Parameters

**DOI:** 10.3390/ani11072078

**Published:** 2021-07-12

**Authors:** Andrea Springer, Daniela Jordan, Alina Kirse, Bettina Schneider, Amely Campe, Gabriela Knubben-Schweizer, Kerstin E. Müller, Martina Hoedemaker, Christina Strube

**Affiliations:** 1 Centre for Infection Medicine, Institute for Parasitology, University of Veterinary Medicine Hannover, 30559 Hanover, Germany; andrea.springer@tiho-hannover.de (A.S.); daniela.jordan@tiho-hannover.de (D.J.); 2 Department of Biometry, Epidemiology and Information Processing (IBEI), WHO Collaborating Centre for Research and Training for Health at the Human-Animal-Environment Interface, University of Veterinary Medicine Hannover, 30559 Hanover, Germany; alina.kirse@tiho-hannover.de (A.K.); bettina.schneider@tiho-hannover.de (B.S.); amely.campe@tiho-hannover.de (A.C.); 3 Clinic for Ruminants with Ambulatory and Herd Health Services, Ludwig-Maximilians-Universität Munich, 85764 Oberschleißheim, Germany; gknubben@med.vetmed.uni-muenchen.de; 4 Clinic for Ruminants and Swine, Faculty of Veterinary Medicine, Free University of Berlin, 14163 Berlin, Germany; Kerstin-Elisabeth.Mueller@fu-berlin.de; 5 Clinic for Cattle, University of Veterinary Medicine Hannover, 30173 Hanover, Germany; martina.hoedemaker@tiho-hannover.de

**Keywords:** *Ostertagia ostertagi*, trichostrongyles, *Fasciola hepatica*, *Dictyocaulus viviparus*, bovine lungworm, bulk tank milk, ELISA, prevalence, milk production, Germany

## Abstract

**Simple Summary:**

Pasture-borne worm infections impact cattle health and productivity worldwide. The present study assessed exposure of dairy cattle herds to the three most important pastural parasites, i.e., gastrointestinal worms, liver flukes and lungworms, in three parts of Germany by measuring antibodies in bulk tank milk samples. The results show a high level of exposure to gastrointestinal worms, while antibodies against liver flukes were less frequently detected and lungworm-positive herds were rare. Regional and breed differences regarding parasite exposure were detected. In addition, the presence of antibodies was associated with access to fresh grass, access to hay, silage quality and deworming frequency. Furthermore, parasite exposure was significantly associated with a poor body condition across all regions. Parasite-exposed cows of high-performance breeds also produced on average less milk per year than dual-purpose breeds.

**Abstract:**

Pasture-borne parasites adversely affect bovine health and productivity worldwide. In Europe, gastrointestinal nematodes, especially *Ostertagia ostertagi*, the liver fluke *Fasciola hepatica* and the lungworm *Dictyocaulus viviparus* represent the most important parasites of dairy cattle. The present study assessed exposure towards these parasites among 646 cattle herds in three parts of Germany during 2017–2019 via antibody detection in bulk tank milk (BTM). Overall, *O. ostertagi* levels indicative of production losses were detected in 41.2% (266/646; 95% confidence interval (CI): 37.4–45.1%) of BTM samples, while *F. hepatica* seroprevalence amounted to 14.9% (96/646; 95% CI: 12.2–17.9%). Only 2.3% (15/646; 95% CI: 1.4–3.9%) of samples were *D. viviparus* antibody-positive. Significantly lower *O. ostertagi* as well as *F. hepatica* seroprevalence was detected in dual-purpose breeds compared to high-performance breeds from the same region. Management factors related to parasite exposure included access to fresh grass and hay, silage quality and anthelmintic treatment. Furthermore, *F. hepatica* and *O. ostertagi* seropositivity was significantly associated with suboptimal herd-level body condition. Interestingly, the relationship between seropositivity and productivity differed between breed types. Negative impacts on milk yield were detected only in high-performance breeds, while *O. ostertagi* seropositivity was associated with a lower milk fat content in dual-purpose herds.

## 1. Introduction

Pasture-borne parasites represent a major global problem for bovine health and productivity. Gastrointestinal nematodes (GIN) of the family Trichostrongylidae, causing parasitic gastroenteritis, are relevant for grazing cattle worldwide [1]. In temperate regions of Europe, including Germany, *Ostertagia ostertagi* is the most prevalent species [2,3,4]. Although infections are often subclinical in dairy cows, studies in several European countries have shown a significant negative correlation between *O. ostertagi* antibody levels and cow productivity, especially in terms of milk yield [5,6]. In addition, a lower milk protein content has been observed in animals with patent GIN infections [7]. The liver fluke *Fasciola hepatica* and the lungworm *Dictyocaulus viviparus* are less prevalent than GIN, but nevertheless represent significant economic burdens [1]. Liver fluke infections mostly cause chronic disease in cattle, resulting in reduced milk yield, impaired fertility, and condemnation of affected livers [8,9,10]. Furthermore, a correlation between elevated β-hydroxybutyrate levels in milk, indicating a negative energy balance and a state of ketosis, and *F. hepatica* antibody titers has been demonstrated [11]. Parasitic bronchitis due to *D. viviparus* may result in severe clinical signs, thus affecting animal welfare and leading to costs for treatment, or even animal mortality [12]. In addition, patent *D. viviparus* infections are associated with a lower average daily milk yield [13]. On herd level, negative effects on milk fat and milk protein content have also been observed [14,15]. 

Screening of bulk tank milk (BTM) samples for the presence of antibodies via ELISA constitutes a reliable and easy method to assess herd parasite exposure [16]. As *O. ostertagi* exposure is generally considered high, available studies usually report mean ELISA optical density ratios (ODRs) rather than prevalence. In Germany, previous studies on dairy cattle using BTM samples indicated mean ODRs of 0.45–0.66, which were regarded intermediate compared to other European countries [17,18]. In this context, BTM ODRs ≥ 0.5 are considered as indicative of a reduction in milk yield [17]. In addition, Fanke et al. [19] reported a seroprevalence of 28.2% using an ODR of 0.6 as cut-off, with 46.5% of herds displaying ODRs between 0.3 and 0.6, and no regional differences. Regarding *F. hepatica*, on average 23.6% of German dairy herds were seropositive in 2008, with considerable regional differences and the highest prevalence rates in the northern and north-western parts of the country [20]. High levels of *F. hepatica* exposure in north-western Germany were also confirmed by recent studies [11,19]. Similar to *F. hepatica*, the lungworm *D. viviparus* shows an unequal distribution in Germany, with regional seroprevalence rates ranging from 0.0% in the south-western federal state of Saarland up to 31.2% in central and northern parts of the country, as determined in the year 2008 [21]. 

Despite the negative consequences of parasite exposure, pasture access for dairy cattle is desirable from an animal-welfare perspective [22] and is increasingly demanded by consumers [23,24]. At the same time, rising levels of anthelmintic resistance and changes in the global climate as well as changes in management practices, e.g., an increase in organic farming systems with a restricted use of anthelmintics, may lead to altered patterns of parasite prevalence in farmed cattle [25]. For example, an increase of *F. hepatica* prevalence among cattle and/or an increase in the geographical spread of this parasite have been observed in some European countries during recent years [26]. Furthermore, a climate-related decrease in *F. hepatica*, but an increase in *O. ostertagi* prevalence was noted over an eight-year study period in Belgium [27]. Similarly, an increase in diagnosed cases of parasitic gastroenteritis as well as dictyocaulosis has been noted from 1975–2014 in the United Kingdom, with more lungworm outbreaks occurring during the winter months [28].

To provide an up-to-date estimate of dairy herd exposure towards GIN (*O. ostertagi*), *F. hepatica* and *D. viviparus*, the current study assessed 646 BTM samples from three different parts of Germany, collected in the period 2017–2019. The dairy industry in these three parts is characterized by distinct structural differences, with medium-sized, numerous farms in northern Germany and numerous, small farms in southern Germany, while fewer, larger farms exist in the eastern part of the country, the former German Democratic Republic [29]. Furthermore, high-performance dairy breeds, such as the Holstein-Friesian, dominate in the northern, western and eastern parts, whereas dual-purpose breeds, such as the German Simmental, are common in southern Germany. Therefore, seroprevalence patterns were analyzed with regard to the different sampling regions and years. Furthermore, regression models were used to assess the impact of management factors on seropositivity and associations with body condition and herd productivity.

## 2. Materials and Methods

### 2.1. Farm Selection, Questionnaire and Farm Visits

In the frame of the “PraeRi” project, a government-funded research project on animal health, biosecurity and housing environment on German dairy cattle farms [30], a total of 8944 farms were initially contacted, of which 765 agreed to participate in the study. Farms were located in three parts of Germany, namely in the North (i.e., the federal states Schleswig-Holstein and Lower Saxony), the East (i.e., the federal states Mecklenburg-Western Pomerania, Brandenburg, Thuringia, Saxony-Anhalt) and in the South (i.e., the federal state of Bavaria). 

Each farm was visited by a team of veterinarians once during the study period. During this visit, the body condition scores (BCS) of cows (up to a maximum of 292 cows/farm) were assessed and categorized according to Edmonson et al. [31] and Metzner et al. [32]. Because body condition changes in a breed-specific manner during lactation, cows were categorized as being below, within and above the optimal BCS range depending on their breed and stage of lactation, as described by Oehm et al. [33]. Additionally, the cow-comfort-quotient according to Nelson [34], skin lesions in different body parts [35,36,37], and lameness [38,39] were assessed. To evaluate the quality of hay and grass silages used by the farmers, samples were taken and analyzed by the service laboratory of the Lower Saxony Chamber of Agriculture (LUFA Nord-West). Using the classification into different LUFA quality scores (QS) ranging from 1 to 4, the farms were categorized as follows: QS < 3: silages with normal to slightly reduced quality (category 0); QS = 3: at least one silage with highly reduced quality (category 1); QS = 4: at least one spoiled silage (category 2). Regarding fully slatted floors, farms were categorized as 1 if at least one compartment for cows had fully slatted floors and 0 if no compartment had fully slatted floors.

During the visit, farmers were interviewed based on an extensive questionnaire with regard to the operational type of the farm, management practices, e.g., access to pasture, feeding regimen and anthelmintic treatment, as well as occurrence of health problems. Based on the interview, the usage of hay in the feeding rations was categorized as follows: low percentage or no hay in the ration (category 0), hay dried differently than on the floor (category 1) and floor-dried hay (category 2). With regard to anthelmintic treatments, different age classes were considered separately: regarding calves (pre-weaning or <6 months of age) and young cattle (weaning/6 months of age to first calving), categories corresponded to no anthelmintic treatment (category 0), treatment of young cattle as well as calves (category 1), treatment of calves only (category 2) and treatment of young cattle only (category 3). The information for lactating and dry cows was categorized accordingly, with category 0 for no anthelmintic treatment, category 1 for treatment of lactating and dry cows, category 2 for anthelmintic treatment of lactating cows only, and category 3 representing treatment of dry cows only.

### 2.2. Breed Information and Milk Production Parameters

Animal-level breed information was obtained from the national cattle registration database (HI-Tier, Bavarian State Ministry for Agriculture and Forestry). Breeds were grouped into “high-performance dairy breeds” (HD), including Holstein-Friesian, Brown Swiss, Angler, Jersey and their crosses, and “dual-purpose breeds” (DP), including German Simmental, Pinzgauer, *Deutsches Schwarzbuntes Niederungsrind* (the founder of the modern Holstein breed) and their crosses. Farms were assigned to the category HD or DP if ≥80% of animals were of the respective breed type; otherwise, they were categorized as mixed-breed farms.

Data on milk production were provided by the milk recording systems of the different federal states (*Landeskontrollverbände*). Monthly test-day data were available from the year prior to the farm visit (i.e., based on approximately 11 test-day records). Based on these recordings, herd averages for annual milk yield per cow (kg), milk protein content (%), milk fat content (%), somatic cell count (SCC), lactation number and number of lactating animals were calculated, excluding farms with less than 10 lactating cows.

### 2.3. BTM Sampling

Following the farm visit, one BTM sample was collected per farm. Sampling was conducted primarily from August–October of 2017, 2018 and 2019, i.e., towards the end of the grazing season, as *D. viviparus* as well as *O. ostertagi* BTM antibody titers reach the highest levels during these months [40,41]. Furthermore, a few samples taken in November were also included. BTM samples were collected in tubes containing a lyophilized bacteriostat (Exactobac-L, nerbe plus GmbH & Co. KG, Winsen/Luhe, Germany) and sent to the Institute for Parasitology, University of Veterinary Medicine Hannover, where they were centrifuged at 2000× *g* for 15 min. The superficial fat layer was removed, and the skimmed milk was stored at −20 °C until analysis.

### 2.4. ELISA Analyses

Samples were run in duplicates in all ELISA analyses. For determination of anti-*O. ostertagi* antibodies, a commercial ELISA kit based on crude adult worm extract was used according to the manufacturer’s instructions (SVANOVIR^®^ *O. ostertagi*-Ab, Boehringer Ingelheim Svanova, Uppsala, Sweden). Results from this test are expressed as optical density ratios (ODRs), with ODRs ≥ 0.5 identifying herds likely to suffer from a negative impact on herd milk yield (infection category +) and ODRs ≥ 0.8 identifying herds likely to suffer substantial production loss due to ostertagiosis (infection category ++) [17,42]. 

Antibodies against the *F. hepatica* f2 antigen were measured using a commercial ELISA test kit as described by the manufacturer (IDEXX Fasciolosis Verification Test, IDEXX GmbH, Kornwestheim, Germany). In this test, results are obtained by comparing the net extinction (NE) of the sample with the NE of the positive control, yielding a sample/positive control ratio (S/P). According to the manufacturer, test results correlate with in-herd prevalence as follows: S/P ≤ 30%: no or very low infection; 30% < S/P ≤ 80%: low infection (proportion of <20% within the herd infected) (infection category +); 80% < S/P < 150%: medium infection (20–50% infected) (infection category ++); S/P ≥ 150%: strong infection (>50% infected) (infection category +++).

An in-house ELISA based on recombinant *D. viviparus* major sperm protein was used to assess antibodies against *D. viviparus* as previously published [40,43,44]. An ODR ≥ 0.41 was considered positive (infection category +) as validated for BTM by Schunn et al. [40].

### 2.5. Data Analyses

All collected data were entered into a central SQL-data base and variables of interest were extracted as Microsoft Excel (Microsoft Corporation, Redmond, WA, USA) spreadsheets. Statistical analyses were conducted using SAS software (Version 9.4 for Windows, SAS Institute Inc., Cary, NC, USA) and R. v. 4.0.2 [45]. 

The distribution of infection categories was compared between the different regions and study years using *χ*^2^-tests (*O. ostertagi*, *F. hepatica*) or Fisher’s Exact tests (*D. viviparus*), respectively. In addition, Fisher’s Exact tests were used to assess breed differences in the distribution of infection categories in the data subset from the southern region. Furthermore, the influence of different predictor variables on *O. ostertagi* and *F. hepatica* seroprevalence was assessed using logistic regression, whereas seroprevalence of *D. viviparus* was too low to conduct a meaningful analysis. In this context, ODRs < 0.5 for *O. ostertagi* were interpreted as negative, and ODRs ≥ 0.5 as positive. Further, S/P-values ≤ 30% for *F. hepatica* were categorized as negative, and S/P-values > 30% as positive. Predictor variables were chosen based on their impact on animal health. The goal was to assess if *O. ostertagi* and *F. hepatica* seroprevalences are lower in herds with improved overall animal health and housing conditions. 

Before building the logistic models, normal distribution for all predictor variables was checked using the Shapiro–Wilk test. Associations between predictor variables were reviewed using Spearman’s correlation coefficient, the Kruskal–Wallis test and Cramér‘s V. The univariate models included “access to fresh grass”, “silage quality”, “anthelmintic treatment of calves and young cattle”, “anthelmintic treatment of lactating and dry cows”, “presence of fully slatted floors” (as an indicator of suboptimal housing), health indicators such as mastitis [%], metritis [%], pneumonia [%], skin lesions [%], BCS [%], the annual average milk yield per cow [kg], the cow-comfort-quotient and lameness [%] as predictor variables. Additionally, access to and drying method of hay was included in the models for *F. hepatica,* since metacercaria can survive the drying process as described previously [46]. The dependent variable “BCS” was defined as the proportion of animals below their optimal BCS range on the farm. Likewise, “mastitis”, “metritis”, “pneumonia”, “skin lesions” and “lameness” were defined as the proportion of animals with the condition. The variables for the multivariate models were selected using a 5% level of significance in the univariate analyses. The multivariate models were built by backward stepwise selection using the -2 Log-Likelihood (-2LL) and a significance level of 20% to estimate the goodness of fit. To assess the effect of predictors on prevalence, the odds ratio (OR) and its 95% confidence interval (CI) were primarily used, considering CIs that did not include 1 as significant, since *p*-values may be unreliable [47]. Because of their presumably large impact on parasite infections, “access to fresh grass”, “anthelmintic treatment of calves and young cattle” and “anthelmintic treatment of lactating and dry cows” were included in the final models regardless of their *p*-value.

Linear regression models (LMs) were used to assess the association between BTM ELISA results and BCS as well as milk production parameters (annual average milk yield per cow [kg], milk protein content [%] and milk fat content [%]) in each study region. Models included the BTM ELISA result category for the three different parasitoses as predictor variables. For BCS, the models contained breed type (HD, DP or mixed) and number of scored animals as further variables. For milk production parameters, breed type, average lactation number, average SCC, average number of lactating animals, and farm type (“conventional”, “organic”, “in transition from conventional to organic”) were included as potential confounders. Due to the expected non-linear relationship, a quadratic term was included for lactation number. As a significant correlation of milk yield with milk fat and protein content was observed, milk yield was included as a potential confounder in these models. Two-way interactions were considered and retained in models if statistically significant (*p* ≤ 0.05). For LM validation, the distribution and homogeneity of model residuals was checked graphically. Cook’s distance and residuals-vs.-leverage plots were used to identify potentially influential observations; these were dropped if their exclusion affected model estimates. Final models were compared to null models containing only an intercept term (R function “anova”).

## 3. Results

### 3.1. Regional and Annual Patterns of Seroprevalence 

**Figure 1:**
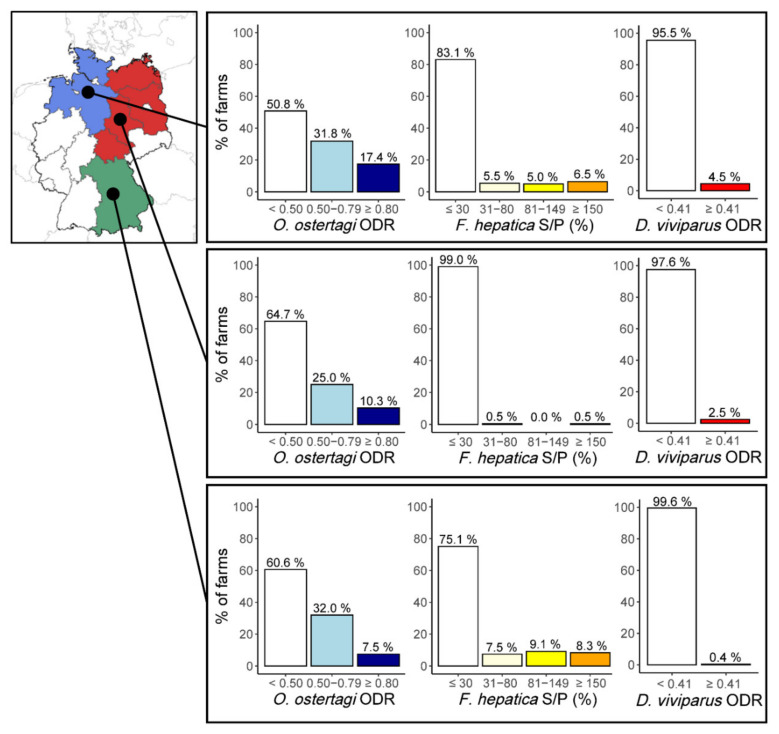
Distribution of *O. ostertagi*, *F. hepatica* and *D. viviparus* antibody level categories as assessed in BTM samples from three different parts of Germany from 2017–2019. The map shows the northern part (i.e., the federal states of Schleswig-Holstein and Lower Saxony) in blue, the eastern part (i.e., the federal states of Mecklenburg-Western Pomerania, Brandenburg, Thuringia, Saxony-Anhalt) in red and the southern part (i.e., the federal state of Bavaria) in green.

Of the 765 participating farms, 646 contributed BTM samples, including 201 from the North, 204 from the East and 241 from the South. Overall, *O. ostertagi* antibody levels indicative of production losses (ODRs ≥ 0.5) were detected in 41.2% (266/646; 95% CI: 37.4–45.1%) of farms, with ODRs between 0.5 and 0.8 in 29.7% (192/646; 95% CI: 26.3–33.4%) and ODRs ≥ 0.8 in 11.5% (74/646; 95% CI: 9.2–14.2%) of farms. Across all samples, the average *O. ostertagi* ODR value was 0.48 (standard deviation [SD]: 0.24). Significant regional differences in the distribution of infection categories were observed (*χ*^2^-test, *χ*^2^ = 15.6, Df = 4, *p* = 0.004), with the highest proportion of farms with ODRs ≥ 0.5 in the northern region (49.3%, 99/201, 95% CI: 42.2–56.4%), followed by the South with 39.4% (95/241, 95% CI: 33.3–45.9%) and the East with 35.3% (72/294, 95% CI: 28.8–42.3%) (Figure 1). In contrast, no significant differences were observed between the study years (*χ*^2^-test, *χ*^2^ = 5.4, Df = 4, *p* = 0.245; Table 1).

Regarding *F. hepatica*, the overall seroprevalence in BTM samples (S/P ≥ 30%) amounted to 14.9% (96/646; 95% CI: 12.2–17.9%), with approximately equal proportions of farms displaying titers indicative of a low (4.6%; 30/646; 95% CI: 3.2–6.6%), medium (5.0%; 32/646; 95% CI: 3.5–7.0%) and high (5.3%; 34/646; 95% CI: 3.7–7.4%) in-herd prevalence. Significant regional differences were observed (*χ*^2^-test, *χ*^2^ = 51.8, Df = 6, *p* < 0.001), with the highest seroprevalence recorded in the South (24.9%, 60/241, 95% CI: 19.7–30.9%), followed by the North (16.9%, 34/201, 95% CI: 12.2–23.0%), while seroprevalence in the East was low (1.0%, 2/204, 95% CI: 0.2–3.9%, Figure 1). Comparison between the different sampling years revealed no significant differences (*χ*^2^-test, *χ*^2^ = 5.9, Df = 6, *p* = 0.434; Table 1). 

Overall *D. viviparus* BTM seroprevalence amounted to 2.3% (15/646; 95% CI: 1.4–3.9%), with significant regional differences (Fisher’s Exact test, *p* < 0.001). The highest seroprevalence was observed in the North (4.5%, 9/201, 95% CI: 2.2–8.6%), followed by the East (2.5%, 5/204, 95% CI: 0.9–5.9%), while seroprevalence in the South was low (0.4%, 1/241, 95% CI: 0.02–2.7%). Similar to the other parasitoses, no significant annual differences were detected (Fisher’s Exact test, *p* = 0.347; Table 1). 

Regarding co-exposure rates, all *D. viviparus*-positive samples also displayed an *O. ostertagi* ODR ≥ 0.5. Furthermore, 85 samples (13.2%, 95% CI: 10.7–16.1%) showed both an *O. ostertagi* ODR of ≥ 0.5 and a positive *F. hepatica* result, with five farms (0.8%, 95% CI: 0.3–1.9%) being additionally *D. viviparus*-positive. Only the co-exposure rate of *O. ostertagi*/*F. hepatica* differed significantly from the numerically expected rate of 6.1% (*χ*^2^ = 17.1, df = 1, *p* < 0.001), indicating a positive association, while no significant differences between the observed and expected values were determined for the other combinations. Patterns of co-exposure in the different sampling regions are shown in Table 2. 

### 3.2. Breed Differences

High-performance dairy cattle breeds dominated in 64.2% (415/646) of farms, whereas 29.1% (188/646) of farms had predominantly DP cattle. A further 5.9% (38/646) of farms had mixed herds, whereas breed information was insufficient for the remaining 0.8% (5/646). 

In the northern and eastern parts of Germany, 97.5% (195/200) and 92.6% (187/202) of farms were assigned to the HD category, respectively. In contrast, 76.6% (183/239) of farms in the South had DP herds, but only 13.8% (33/239) had HD and 9.6% (23/239) had mixed herds. 

**Figure 2:**
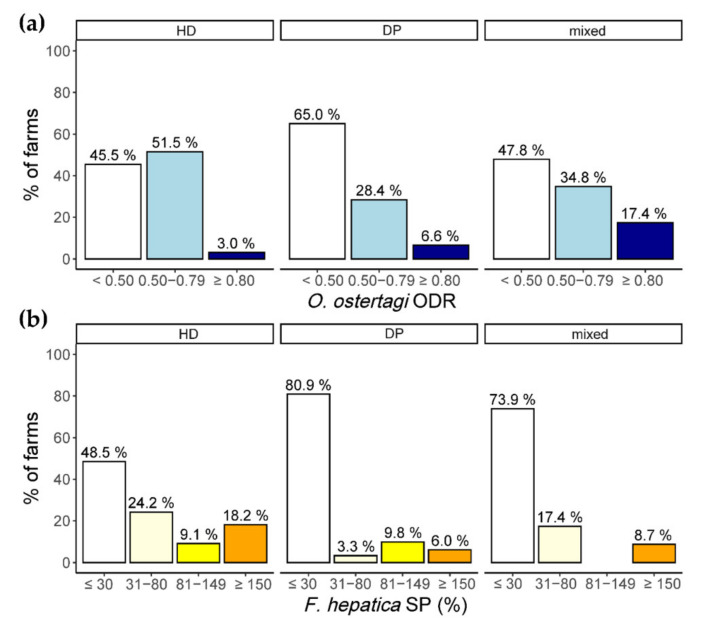
Distribution of *O. ostertagi* (**a**) and *F. hepatica* (**b**) BTM antibody level categories in southern Germany, according to herd breed type. Herds were defined as high-performance dairy (HD) or dual-purpose (DP) if ≥80% of animals belonged to the respective breed type, the remaining herds were defined as “mixed”.

In the South, significant differences in antibody level category distribution were observed regarding *O. ostertagi* and *F. hepatica*, with lower prevalence in DP than HD herds (Fisher-Exact tests, *p* = 0.025 and *p* < 0.001, respectively; Figure 2). Differences were not assessed for *D. viviparus* due to the low prevalence in this region. 

### 3.3. Association of Seroprevalence with Management Factors

Substantial regional differences in herd management were observed for the factors of access to fresh grass, access to hay and anthelmintic treatment (Supplementary Table S1). While 47.8% of herds had access to fresh grass in the North, this applied to only 24.9% of herds in the East and 32.0% in the South. In contrast, less than 10% of farms included hay in rations in the North and East, whereas more than 20% of farms fed hay in the South. Spoiled silages were found in 50.8% of farms in the North, 29.8% of farms in the East and 38.6% of farms in the South. Regarding anthelmintic treatment, 73.0% and 50.8% of farms in the North regularly treated calves/young cattle and dry/lactating cows, respectively, as compared to only 38.5% and 23.1% in the East and 30.5% and 19.5% in the South. 

The following variables were included in the final multivariate logistic regression models for *O. ostertagi* after exploratory univariate analyses: access to fresh grass, silage quality (North only), anthelmintic treatment, lameness (South only) and average annual milk yield per cow (Table 3). Access to fresh grass significantly increased the odds of a BTM ELISA result ≥0.5 in all three regions, with ORs of 5.5 (95% CI: 2.5–12.1) in the North, 3.7 (95% CI: 1.8–7.9) in the East and 7.0 (95% CI: 3.1–15.4) in the South. Furthermore, the use of spoiled silages increased the odds for a positive result by 3.0 (95% CI: 1.4–6.5) in the North. Anthelmintic treatment of young cattle was positively associated with *O. ostertagi* BTM seroprevalence in the North and in the South, while treatment of both calves and young cattle was positively associated with *O. ostertagi* status in the South only (Table 3). The association of seropositivity with average annual milk yield per cow was significant in all three regions. Therefore, the impact of *O. ostertagi* exposure on milk production was further investigated in detail using linear regression (see below).

With regard to *F. hepatica*, access to fresh grass, access to hay (South only), anthelmintic treatment, lameness (South only) and annual average milk yield were included in the final multivariate models. Risk factors for *F. hepatica* could not be identified in the North and were not investigated in the East due to low prevalence. In the South, however, access to fresh grass, access to hay and the treatment of calves and young cattle with anthelmintics were positively associated with *F. hepatica* seropositivity, whereas a negative relationship was detected regarding the average annual milk yield per cow as well as lameness (Table 4). 

### 3.4. Association of Seropositivity Categories with BCS and Herd Productivity Parameters 

**Figure 3:**
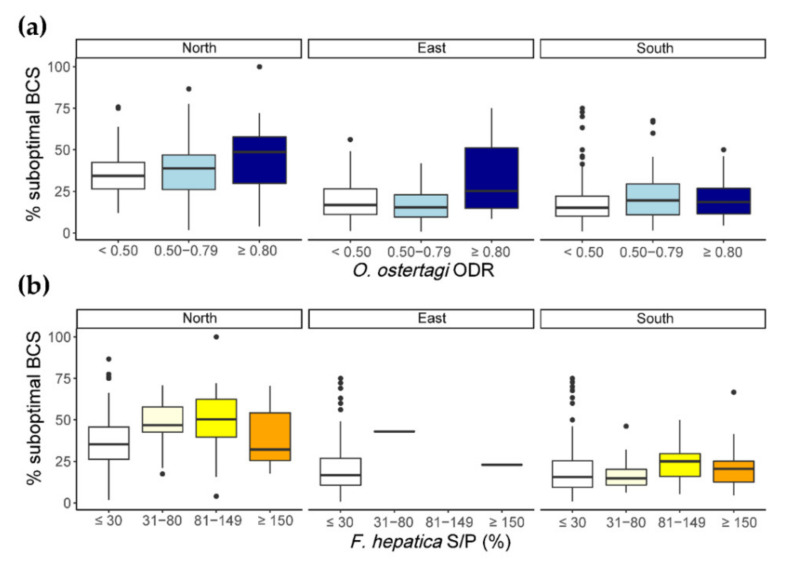
Percentage of cows with a suboptimal body condition score (BCS) according to BTM antibody level categories for *O. ostertagi* (**a**) and *F. hepatica* (**b**).

In all three regions, the proportion of cows displaying a suboptimal BCS was significantly associated with BTM ELISA results (Table 5, Figure 3). In the North and the South, this proportion was significantly increased on farms with both positive *O. ostertagi* (category ++) and *F. hepatica* antibody levels (category ++) compared to seronegative farms, respectively. In addition, the proportion of cows displaying a suboptimal BCS was also significantly increased in herds with an *O. ostertagi* category ++ and a *F. hepatica* category + antibody level in the South. In the East, where almost no farms were *F. hepatica*-seropositive, a significantly higher proportion of BCS-suboptimal cows was found on farms with an *O. ostertagi* ODR ≥ 0.8 (category ++). However, the effect of infection on BCS was not consistent across all antibody level categories, as a significantly lower proportion of suboptimal cows was present on farms with a *F. hepatica* (category +) status in the South and *O. ostertagi* (+)/*F. hepatica* (+++) status in the North, as compared to seronegative farms. Additionally, breed type was significantly associated with BCS, with a lower proportion of suboptimal cows in DP herds (Table 5). However, no indication of a breed difference regarding the effect of parasite exposure on BCS was found (data not shown). Finally, the number of scored cows had a small but significant negative effect regarding the proportion of suboptimal cows.

Herd productivity parameters were available for 596 farms (192 from the North, 196 from the East, 208 from the South). A significant negative impact of *F. hepatica* seropositivity on milk yield was detected in the northern region, with an estimated annual reduction of 1129.2–1335.3 kg milk/cow in seropositive vs. seronegative herds, depending on seropositivity category (Table 6). In the East, where *F. hepatica* seroprevalence was low, a significant negative effect of *O. ostertagi* seropositivity was apparent, with an estimated reduction in annual milk yield of 832.7 kg/cow in herds with an ODR ≥ 0.8 (category ++). 

As the model for the southern part of Germany indicated a significant interaction of breed type and *F. hepatica* BTM result (Table 6), separate models were additionally calculated for HD and DP herds. A significant negative impact of *F. hepatica* seropositivity (infection category +++) on milk yield was only detected in HD, but not in DP herds, which instead displayed a significantly higher milk yield in the *F. hepatica* category +++ (Supplementary Table S2). Further factors significantly associated with milk yield were breed type, lactation number, SCC, number of lactating animals and farm type (Table 6, Supplementary Table S2). Regarding *D. viviparus*, no significant impact on milk yield was detected in northern and eastern Germany. The dataset from the South, for which milk production data were available, contained no *D. viviparus*-positive herds.

Regarding average milk protein content (%), the number of producing animals had no significant effect on milk protein and removing this variable significantly improved model fit. Likewise, removing the variable “milk yield” from the model for the North led to an improved model. Only farm type had a significant effect in all three datasets, while significant differences between breed types were noted in the East and in the South (Table 7). A significant association of BTM results with milk protein was only detected regarding *F. hepatica* category ++ in the North, with a positive effect (Table 7). For the southern region, separate models were again calculated for HD and DP herds, and only the HD model suggested a negative association of *F. hepatica* infection category +++ with protein content (Supplementary Table S2), however, the model was not significantly different from a null model and thus needs to be considered of limited reliability. 

Average milk fat content (%) was significantly negatively associated with milk yield in all three regions (Table 8). In contrast, a significant association with *O. ostertagi* BTM ELISA results was only noted in the South, where herds with an ODR ≥ 0.8 had significantly lower average fat content (Table 8). This was primarily driven by DP herds, as the dataset did not contain any HD herds in this antibody level category (Supplementary Table S1).

## 4. Discussion

The aim of the current study was to provide an up-to-date estimate of dairy cow exposure to *O. ostertagi, F. hepatica* and *D. viviparus* in three parts of Germany, which show considerable structural differences in dairy farming, and to assess relationships with management factors as well as production parameters. 

The overall mean *O. ostertagi* ODR of 0.48 is similar to previous investigations and can be considered intermediate in comparison to other European countries, where mean ODRs as low as 0.31 and as high as 0.83 have been measured [17,18]. In contrast to Fanke et al. [19], who reported no significant regional differences, the current study demonstrated a higher level of *O. ostertagi* exposure in the northern as compared to the eastern and the southern study areas. This can be explained by the high proportion of farms which provided access to fresh grass in this area. In all three datasets, access to fresh grass was significantly associated with *O. ostertagi* exposure, in line with the results of previous studies [17,48,49]. Furthermore, the presence of spoiled silage on the farm increased the odds for *O. ostertagi* BTM ODRs ≥ 0.5 in the North. Possibly, spoiled silage may negatively affect the overall health status of cows and their susceptibility towards GIN infections. In addition, anthelmintic treatment of young cattle was positively associated with *O. ostertagi* seropositivity in the North and the South, probably because farms with known GIN problems increase their use of anthelmintics. Frequent use of anthelmintics in young animals, however, impairs the development of an efficacious immune response, rendering them more susceptible to *O. ostertagi* as adults [50].

The overall *F. hepatica* seroprevalence of 14.9% determined in the current study is lower than previously determined values for German dairy herds of 23.6% [20] and 22.9% [19]. This is mainly driven by a lower *F. hepatica* seroprevalence (16.9%) in northern Germany as compared to previous investigations. In samples collected in 2008, Kuerpick et al. [20] determined *F. hepatica* BTM seroprevalences of 29.4–38.4% in those federal states, which were included in the northern region in the present study. Indeed, a spatial model of fasciolosis in dairy cattle, including meteorological factors, identified the northwestern part of Germany as a high-risk area, but indicated a low risk in eastern and southern parts of Germany [51]. However, 2018 and 2019 were exceptionally dry years in central Europe [52], which may have led to unfavorable conditions for the snail intermediate host of *F. hepatica*, which depends on moist habitats. Thus, the decline in *F. hepatica* seroprevalence may be related to climate change. In contrast, May et al. [11] observed high *F. hepatica* seroprevalences of 33.1–37.0% during 2017–2018 in East Frisia, a coastal area of Lower Saxony, employing the same serological test as the current study. However, the landscape of East Frisia is characterized by coastal marshlands traversed by drainage channels and was probably less affected by the dry weather. In addition, farmers received feedback regarding the BTM ELISA results of the previous studies, so the decline in seroprevalence observed in the present study may be due to effective *F. hepatica* control measures [11]. Furthermore, the geographical distribution of the farms within the northern region may be responsible for the observed discrepancies, or the study design may have created a bias since it was based on voluntary participation by farmers. In cases of known problems with fasciolosis on the farm, farmers may have chosen not to participate. Furthermore, it is possible that the increased use of anthelmintics in calves and young cattle as well as in lactating and dry cows in the northern region in the current study led to a lower prevalence.

In contrast, the low *F. hepatica* seroprevalence of 1% in the East is in accordance with previous observations [20]. For southern Germany, a heterogeneous distribution of *F. hepatica* has been described, with high levels of exposure in Alpine regions, but low levels in the Bavarian plains [53]. The overall seroprevalence of 24.9% is similar to the value of 32.2% determined by Koch in 2005 [53]; however, the data are not completely comparable due to different cut-offs used to define seropositivity. Interestingly, a significantly lower seroprevalence of *F. hepatica* as well as *O. ostertagi* was determined in DP as compared to HP dairy herds in the South. This might be driven by differences in animal husbandry. Alternatively, DP breeds might have higher liver fluke resistance than HP dairy breeds, which have often been selected for indoor production systems. May et al. [24] determined a higher level of resistance against GINs in two Holstein-Friesian lines selected for grazing systems as compared to lines selected for indoor production. However, similar studies comparing Holstein-Friesian to German DP breeds with regard to liver flukes are not available so far. 

Previous research in temperate climatic zones has shown that management factors, such as treatment with flukicides, may have a larger impact on herd infection status with *F. hepatica* than climatic variables [42,43]. In the present study, anthelmintic treatment in calves and young cattle was positively related to *F. hepatica* exposure in Bavaria, probably because farms with known fasciolosis problems practice frequent treatment. Furthermore, access to fresh grass increased the odds of a positive *F. hepatica* BTM ELISA result, in line with previous studies [49]. This demonstrates that pasture and feeding management remain key factors in decreasing parasite exposure. Since metacercaria of *F. hepatica* can survive in hay for up to six months, depending on the degree of desiccation [46], access to hay was included in the analysis. A significant impact of this variable was only found in the dataset from the South. In this part of Germany, the proportion of farms feeding hay was larger than in the other parts, whereas less farms provided access to fresh grass. Finally, the percentage of cows with lameness was negatively associated with herd seropositivity in the South. Further studies are needed to shed light on the underlying reasons and possible confounding factors, especially since the occurrence of lameness was distributed evenly in all three regions (data not shown). 

Seroprevalence values for *D. viviparus* among German dairy herds determined in the year 2008, using the same serologic test as the current study, ranged from 0.0–31.2% in different regions, with an overall value of 17.1% [21]. In comparison to the dataset from 2008, both the overall *D. viviparus* seroprevalence of 2.3%, as well as the regional seroprevalence values determined in the current study were surprisingly low, as rates of 10.4–31.2% were determined by Schunn et al. [21] in the regions relevant for the current study. As mentioned above, with regard to *F. hepatica*, farmers were informed about the results of these studies, and may have changed their management practices, resulting in the observed prevalence decline. Unfortunately, *D. viviparus* herd seroprevalence in Germany has not been assessed between 2008 and 2017, hampering the interpretation of our data. Alternatively, the decline in seroprevalence may reflect endemic stability, as the magnitude and duration of the antibody response are reduced in repeatedly infected cows, resulting in lower BTM ODRs [54]. Therefore, further studies are necessary to determine whether *D. viviparus* may be on the decline in Germany, e.g., due to increasingly dry and warm summers as observed especially in the years 2018 and 2019 [52], or whether other factors are responsible for the low seroprevalence in the current study. As mentioned regarding *F. hepatica*, the study design may have caused a bias, as farms with a dictyocaulosis history may have been less likely to participate. Given the low seroprevalence of *D. viviparus*, risk factor analysis was not possible in the present study. In previous studies, herd size, mowing of pastures and length of the grazing period were associated with *D. viviparus* antibody status [15,55].

The present study determined a significant association of parasite exposure with health indicators and cow productivity. *O. ostertagi* and *F. hepatica* exposure were significantly associated with a low herd-level body condition in all three datasets. In the North as well as the South, herds with evidence of co-exposure to both parasites had a significantly higher proportion of cows in suboptimal condition. In the East, where *F. hepatica* seroprevalence was very low, a significantly higher proportion of thin cows was found on farms with an *O. ostertagi* BTM ODR ≥ 0.8. Similarly, *Ostertagia* spp. and *F. hepatica* infected beef cattle displayed lower carcass weights than helminth-free animals in the United Kingdom [3]. Additionally, liver fluke infections in dairy cows have been associated with higher levels of beta-hydroxybutyrate in milk, indicating a negative energy balance [11]. Therefore, the current study contributes to the evidence that helminth infections do not only lead to subclinical impacts on dairy cow productivity, but may also contribute to a low body condition, which entails further health risks [56]. 

In line with previous studies, a negative association between *O. ostertagi* and *F. hepatica* exposure and milk yield was demonstrated in the current analysis, although not to the same extent in all regions. Similar to the pattern regarding BCS, a significant association of *O. ostertagi* exposure and reduced milk yield was only detected in the East, where a loss of 832.74 kg/milk per cow and year was estimated in herds with a BTM ODR ≥ 0.8. This value is similar to an estimated loss of 975 litres of milk per cow and year in *O. ostertagi*-seropositive herds in a previous German study [19]. In the other study areas, *F. hepatica* rather than *O. ostertagi* exposure was significantly associated with a reduced milk yield, with estimated losses of more than 1000 kg milk/cow and year. Interestingly, breed differences seem to play an important role regarding regional differences. In the South, where DP breeds (mainly German Simmental) are common, a negative association between milk yield and *F. hepatica* exposure was only detected in herds consisting of HD breeds. Therefore, DP breeds not only had lower parasite seroprevalences in the current study, but also seem to be more resilient towards negative effects of parasite infection on milk yield. Similar differences in resilience exist among sheep and goat breeds with a moderately heritable genetic basis, and have been suggested for cattle [57,58]. These breeds may thus be especially suited for organic farming, where “robust” breeds are preferred due to restricted drug use, including anthelmintics [57]. However, DP herds in the South showed a significantly lower milk fat content associated with *O. ostertagi* seropositivity, while this was not observed in HD breeds in the current study, nor in previous investigations [5,7]. Therefore, resilience may be limited to milk yield, whereas GIN infection may adversely affect milk fat content in DP breeds, which should be confirmed in further studies, preferably on an individual animal basis. A drop in milk fat content due to *F. hepatica* infections as observed in previous studies [9,11,59] was not noted in the present dataset. 

Furthermore, no significant association of parasite seroprevalence with milk protein content was found, except for a 0.1% higher average protein content in *F. hepatica* category ++ vs. seronegative herds in the North. Most previous studies found either no [8,59] or a significant negative association between *F. hepatica* infections and milk protein content [9,11]. Therefore, the estimated increase in milk protein in this group of cattle may be due to an unknown confounding factor. 

## 5. Conclusions

The current study indicated that *O. ostertagi* and *F. hepatica* exposure among dairy herds seems to be rather stable in Germany, while further studies are needed to assess whether *D. viviparus* is indeed on the decline. Management factors related to parasite exposure included access to fresh grass and hay, silage quality and anthelmintic treatment, with some regional differences related to variation in animal husbandry. The significant differences between cattle breed types with regard to parasite seroprevalence as well as impacts on production parameters represent a novel finding. Lower prevalence of *O. ostertagi* and *F. hepatica* in dual-purpose herds and their increased resilience in terms of unaffected milk yield indicate that this breed type may be especially suited for pasture-based or organic settings, if these findings are confirmed in further studies.

## Figures and Tables

**Table 1:** Annual variation in seroprevalence of major pasture-borne parasitoses in BTM samples from German dairy cattle.

	2017		2018		2019	
	% (Pos./Total)	95% CI	% (Pos./Total)	95% CI	% (Pos./Total)	95% CI
***O. ostertagi*^a^**	36.5 (73/200)	29.9–43.6%	41.7 (98/235)	35.4–48.3%	45.0 (95/211)	38.2–52.0%
***F. hepatica***	15.0 (30/200)	10.5–20.9%	11.5 (27/235)	7.8–16.4%	18.5 (39/211)	13.6–24.5%
***D. viviparus***	2.5 (5/200)	0.9–6.0%	1.3 (3/235)	0.3–4.0%	3.3 (7/211)	1.5–7.0%

^a^ *O. ostertagi*-positive defined as ODR ≥ 0.5.

**Table 2:** Patterns of parasite co-exposure in 646 BTM samples from three different regions of Germany.

	North		East		South	
	% (pos./total)	95% CI	% (pos./total)	95% CI	% (pos./total)	95% CI
***O. ostertagi*** ^a^ **& *F. hepatica***	14.9% (30/201)	10.4–20.8%	0.1% (1/204)	0.0–3.1%	22.4% (54/241)	17.4–28.3%
***O. ostertagi*** ^a^ **& *D. viviparus***	4.5% (9/201)	2.2–8.6%	2.5% (5/204)	0.9–5.9%	0.4% (1/241)	0.02–2.7%
***O. ostertagi*** ^a^ **& *F. hepatica* &** ***D. viviparus*** ^b^	2.0% (4/201)	0.6–5.3%	0.0% (0/204)	0.0–2.3%	0.4% (1/241)	0.02–2.7%

^a^*O. ostertagi*-positive defined as ODR ≥ 0.5. ^b^ All samples positive for both *F. hepatica* and *D. viviparus* were also *O. ostertagi*-positive.

**Table 3:** Results from multivariate logistic models investigating the influence of predictor variables on *O. ostertagi* BTM ELISA results (North: Df = 172; East: Df = 183; South: Df = 190). Significant ORs and CIs are printed in bold.

	North						East						South					
Variable	OR	95% CI	-2LL	nDF	F	*p*	OR	95% CI	-2LL	nDF	F	*p*	OR	95% CI	-2LL	nDF	F	*p*
**Access to fresh grass**	-	-	185.90	1	18.47	<0.001	-	-	207.85	1	11.86	<0.001	-	-	179.54	1	23.11	<0.001
Yes vs. no	**5.53**	**2.52–12.12**	-	-	-	-	**3.73**	**1.75–7.94**	-	-	-	-	**6.96**	**3.14–15.42**	-	-	-	-
**Silage quality**	-	-	185.90	2	5.47	0.005	-	-	-	-	-	-	-	-	-	-	-	-
Lower vs. normal	0.43	0.07–2.54	-	-	-	-	-	-	-	-	-	-	-	-	-	-	-	-
Spoiled vs. normal	**3.03**	**1.42–6.48**	-	-	-	-	-	-	-	-	-	-	-	-	-	-	-	-
**Anthelmintic treatment Calves & young cattle**	-	-	185.90	2	2.33	0.100	-	-	207.85	2	1.42	0.245	-	-	179.54	2	2.98	0.053
Both vs. no treatment	1.45	0.41–5.16	-	-	-	-	3.16	0.34–29.16	-	-	-	-	**4.65**	**1.01–21.40**	-	-	-	-
Yc vs. no treatment	2.59	1.00–6.71	-	-	-	-	1.84	0.84–4.02	-	-	-	-	**2.89**	**1.08–7.78**	-	-	-	-
**Anthelmintic treatment** **Lactating & dry cows**	-	-	185.90	3	1.28	0.281	-	-	207.85	3	1.56	0.200	-	-	179.54	2	0.26	0.771
Both vs. no treatment	1.00	0.44–2.24	-	-	-	-	0.26	0.06–1.07	-	-	-	-	1.12	0.33–3.74	-	-	-	-
Lc vs. no treatment	4.34	0.92–20.45	-	-	-	-	1.52	0.31–7.59	-	-	-	-	-	-	-	-	-	-
Dc vs. no treatment	0.80	0.10–6.16	-	-	-	-	1.50	0.37–6.01	-	-	-	-	2.08	0.28–15.27	-	-	-	-
**Lameness**	-	-	-	-	-	-	-	-	-	-	-	-	-	-	179.54	1	1.92	0.167
10% increase from mean	-	-	-	-	-	-	-	-	-	-	-	-	0.82	0.62–1.09	-	-	-	-
**Milk yield**	-	-	185.90	1	6.08	0.015	-	-	207.85	1	10.88	0.001	-	-	179.54	1	13.08	<0.001
500 kg increase from mean	**0.83**	**0.72–0.96**	-	-	-	-	**0.82**	**0.73–0.92**	-	-	-	-	**0.75**	**0.64–0.88**	-	-	-	-

Abbreviations: -2LL, -2 Log-Likelihood; nDf, numeric degrees of freedom; F, F-Value; OR, odds ratio; CI, confidence interval; yc, young cattle; lc, lactating cows; dc, dry cows.

**Table 4:** Results from multivariate logistic models investigating the influence of predictor variables on *F. hepatica* BTM ELISA results (North: Df = 175; South: Df = 186). Significant ORs and CIs are printed in bold.

	North						South					
Variable	OR	95% CI	-2LL	nDF	F	*p*	OR	95% CI	-2LL	nDF	F	*p*
**Access to fresh grass**	-	-	125.87	1	3.50	0.063	-	-	104.02	1	15.52	<0.001
Yes vs. no	2.74	0.95–7.92	-	-	-	-	**8.71**	**2.95–25.75**	-	-	-	-
**Hay**	-	-	-	-	-	-	-	-	104.02	2	4.78	0.009
Floor-dried vs. no hay	-	-	-	-	-	-	**12.19**	**1.21–122.50**	-	-	-	-
Dried otherwise vs. no hay	-	-	-	-	-	-	**4.97**	**1.46–16.87**	-	-	-	-
**Anthelmintic treatment** **Calves & young cattle**	-	-	125.87	2	1.54	0.218	-	-	104.02	2	8.61	<0.001
Both vs. no treatment	5.04	0.49–52.13	-	-	-	-	19.42	3.07–122.75	-	-	-	-
Yc vs. no treatment	6.58	0.76–56.76	-	-	-	-	16.57	4.20–65.43	-	-	-	-
**Anthelmintic treatment** **Lactating & dry cows**	-	-	125.87	3	0.40	0.753	-	-	104.02	2	4.39	0.014
Both vs. no treatment	0.96	0.35–2.63	-	-	-	-	0.24	0.06–1.02	-	-	-	-
Lc vs. no treatment	1.05	0.21–5.34	-	-	-	-	-	-	-	-	-	-
Dc vs. no treatment	2.62	0.41–16.83	-	-	-	-	8.37	0.94–74.94	-	-	-	-
**Lameness**	-	-	-	-	-	-	-	-	104.02	1	6.69	0.010
10% increase from mean	-	-	-	-	-	-	**0.58**	**0.38–0.88**	-	-	-	-
**Milk yield**	-	-	125.87	1	-	<0.001	-	-	104.02	1	5.92	0.016
500 kg increase from mean	0.79	-	-	-	-	-	**0.75**	**0.60–0.95**	-	-	-	-

Abbreviations: -2LL, -2 Log-Likelihood; nDf, numeric degrees of freedom; F, F-Value; OR, odds ratio; CI, confidence interval; yc, young cattle; lc, lactating cows; dc, dry cows.

**Table 5:** Results from linear models investigating the association of *O. ostertagi*, *F. hepatica* and *D. viviparus* BTM ELISA results and the proportion of cows with a suboptimal body condition score. Full models were significantly different from null models containing only an intercept term (North: Df = 14, F = 4.1, *p* < 0.001; East: Df = 8, F = 7.1, *p* < 0.001; South: Df = 13, F = 4.2, *p* < 0.001). Significant *p*-values are printed in bold. Seropositivity categories are defined as follows: *O. ostertagi*: +: ODR ≥ 0.5, ++: ODR ≥ 0.8; *F. hepatica*: +: 30% < S/P ≤ 80%, ++: 80% < S/P < 150%, +++: S/P ≥ 150%; *D. viviparus*: +: ODR ≥ 0.41.

	North (N = 199) ^a^				East (N = 197)				South (N = 230)			
Variable	Est.	SE	*t*	*p*	Est.	SE	*t*	*p*	Est.	SE	*t*	*p*
Intercept	0.42	0.03	15.02	**<0.001**	0.29	0.02	11.77	**<0.001**	0.37	0.04	10.08	**<0.001**
*O. ostertagi* +	0.02	0.03	0.88	0.382	−0.02	0.02	−1.18	0.241	0.03	0.02	1.13	0.261
*O. ostertagi* ++	−0.01	0.04	−0.28	0.778	0.11	0.03	3.52	**0.001**	−0.11	0.07	−1.74	0.083
*F. hepatica* +	−1.48 × 10^−3^	0.10	−0.01	0.989	0.18	0.12	1.51	0.133	−0.14	0.07	−2.01	**0.046**
*F. hepatica* ++	−0.04	0.10	−0.39	0.700	-	-	-	-	−0.20	0.13	−1.50	0.135
*F. hepatica* +++	0.11	0.07	1.64	0.103	−0.08	0.09	−0.95	0.345	0.02	0.09	0.23	0.820
*O. ostertagi* +/*F. hepatica* +	0.03	0.13	0.26	0.793	-	-	-	-	−0.01	0.08	−0.12	0.903
*O. ostertagi* ++/*F. hepatica* +	0.18	0.13	1.33	0.187	-	-	-	-	0.32	0.12	2.75	**0.006**
*O. ostertagi* +/*F. hepatica* ++	0.13	0.15	0.88	0.381	-	-	-	-	0.18	0.14	1.31	0.193
*O. ostertagi* ++/*F. hepatica* ++	0.33	0.13	2.59	**0.010**	-	-	-	-	0.43	0.16	2.72	**0.007**
*O. ostertagi* +/*F. hepatica* +++	−0.20	0.09	−2.18	**0.030**	-	-	-	-	−0.05	0.10	−0.55	0.580
*O. ostertagi* ++/*F. hepatica* +++	-	-	-	-	-	-	-	-	-	-	-	-
*D. viviparus* +	0.10	0.06	1.74	0.084	−0.07	0.06	−1.26	0.209	-	-	-	-
Breed type (DP vs. HD)	−0.37	0.11	−3.53	**0.001**	−0.03	0.09	−0.32	0.750	−0.10	0.03	−3.63	**<0.001**
Breed type (Mix vs. HD)	−0.02	0.11	−0.20	0.842	−0.06	0.04	−1.67	0.097	−0.08	0.04	−2.26	**0.025**
No. of scored cows	−5.82 × 10^−4^	2.20 × 10^−4^	−2.65	**0.009**	−4.22 × 10^−4^	9.83 × 10^−5^	−4.29	<0.001	−1.75 × 10^−3^	3.54 × 10^−4^	−4.95	**<0.001**

^a^ One outlier was excluded. Abbreviations: Est., estimate; DP, dual-purpose; HD, high-performance dairy; SE, standard error.

**Table 6:** Results from linear models investigating the association of *O. ostertagi*, *F. hepatica* and *D. viviparus* BTM ELISA results and average annual milk yield per cow (kg) in three regions of Germany. Full models were significantly different from null models containing only an intercept term (North: Df = 13, F = 8.5, *p* < 0.001; East: Df = 11, F = 20.1, *p* < 0.001; South: Df = 18, F = 8.8, *p* < 0.001). Significant *p*-values are printed in bold. Seropositivity categories are defined as follows: *O. ostertagi*: +: ODR ≥ 0.5, ++: ODR ≥ 0.8; *F. hepatica*: +: 30% < S/P ≤ 80%, ++: 80% < S/P < 150%, +++: S/P ≥ 150%; *D. viviparus*: +: ODR ≥ 0.41.

	North (N = 192)				East (N = 193) ^a^				South (N = 207) ^b^			
Variable	Est.	SE	*t*	*p*	Est.	SE	*t*	*p*	Est.	SE	*t*	*p*
Intercept	6441.89	1985.87	3.24	**0.001**	−167.19	2605.5	−0.06	0.949	9611.54	1590.34	6.0	**<0.001**
*O. ostertagi* +	−153.97	196.78	−0.78	0.435	−195.30	193.75	−1.01	0.315	−219.67	188.72	−1.16	0.246
*O. ostertagi* ++	−40.11	312.66	−0.13	0.898	−832.74	326.22	−2.55	**0.012**	−403.61	346.48	−1.17	0.246
*F. hepatica* +	−1157.71	372.17	−3.11	**0.002**	1416.02	1106.52	1.28	0.202	132.62	469.94	0.28	0.778
*F. hepatica* ++	−1335.25	445.64	−3.00	**0.003**	-	-	-	-	−273.37	316.56	−0.86	0.389
*F. hepatica* +++	−1129.20	376.19	−3.00	**0.003**	-	-	-	-	985.30	355.25	2.77	**0.006**
*D. viviparus* +	159.61	430.40	0.37	0.711	724.11	509.20	1.42	0.157	-	-	-	-
Breed type (DP vs. HD)	−2236.55	855.00	−2.62	**0.010**	−1576.49	785.00	−2.01	**0.046**	−1259.64	293.53	−4.29	**<0.001**
Breed type (Mix vs. HD)	721.34	861.16	0.84	0.403	−1015.21	357.5	−2.84	**0.005**	−1399.50	370.78	−3.77	**<0.001**
Lactation no.	2723.21	1330.63	2.05	**0.042**	7844.89	1847.15	4.25	**<0.001**	−289.28	1037.87	−0.28	0.781
Lactation no. (squared)	−504.38	225.54	−2.24	**0.027**	−1458.48	328.97	−4.43	**<0.001**	52.26	166.83	0.31	0.754
SCC (×1000/mL)	−4.27	1.06	−4.01	**<0.001**	−2.63	0.90	−2.93	**0.004**	−2.77	0.85	−3.26	**0.001**
No. of animals	3.92	1.71	2.29	**0.023**	0.59	0.23	2.50	**0.013**	7.95	2.57	3.10	**0.002**
farm type (ORG vs. CON)	−1522.86	522.11	−2.92	**0.004**	−2446.93	343.01	−7.13	**<0.001**	−1685.37	266.41	−6.33	**<0.001**
farm type (TRA vs. CON)	-	-	-	-	-	-	-	-	−245.64	399.71	−0.62	0.540
Breed (DP)/*F. hepatica* +	-	-	-	-	-	-	-	-	759.10	648.05	1.17	0.243
Breed (Mix)/*F. hepatica* +	-	-	-	-	-	-	-	-	498.60	868.11	0.57	0.566
Breed (DP)/*F. hepatica* ++	-	-	-	-	-	-	-	-	−2.10	692.58	−0.00	0.998
Breed (Mix)/*F. hepatica* ++	-	-	-	-	-	-	-	-	-	-	-	-
Breed (DP)/*F. hepatica* +++	-	-	-	-	-	-	-	-	1430.93	685.62	2.09	**0.038**
Breed (Mix)/*F. hepatica* +++	-	-	-	-	-	-	-	-	2050.29	1128.19	1.82	0.071

^a^ Three outliers were excluded, these were the three smallest herds in the northeastern region. ^b^ One outlier was excluded, this was the largest herd in the Bavarian dataset. Abbreviations: CON, conventional; Est., estimate; DP, dual-purpose; HD, high-performance dairy; ORG, organic; SCC, somatic cell count; SE, standard error; TRA, in transition from conventional to organic.

**Table 7:** Results from linear models investigating the association of *O. ostertagi*, *F. hepatica* and *D. viviparus* BTM ELISA results and average milk protein content (%) in three regions of Germany. Full models were significantly different from null models containing only an intercept term (North: Df = 12, F = 2.3, *p* = 0.009; East: Df = 14, F = 2.7, *p* = 0.001; South: Df = 15, F = 6.0, *p* < 0.001). Significant *p*-values are printed in bold. Seropositivity categories are defined as follows: *O. ostertagi*: +: ODR ≥ 0.5, ++: ODR ≥ 0.8; *F. hepatica*: +: 30% < S/P ≤ 80%, ++: 80% < S/P < 150%, +++: S/P ≥ 150%; *D. viviparus*: +: ODR ≥ 0.41.

	North (N = 192)				East (N = 195) ^a^				South (N = 208)			
Variable	Est.	SE	*t*	*p*	Est.	SE	*t*	*p*	Est.	SE	*t*	*p*
Intercept	3.58	0.15	24.03	**<0.001**	3.78	0.22	17.11	**<0.001**	4.08	0.25	16.58	**<0.001**
*O. ostertagi* +	−0.01	0.01	−0.87	0.388	0.01	0.02	0.43	0.666	4.36 × 10^−3^	0.02	0.21	0.831
*O. ostertagi* ++	2.81 × 10^−3^	0.02	0.12	0.902	0.01	0.03	0.22	0.823	0.01	0.04	0.18	0.861
*F. hepatica* +	0.02	0.03	0.56	0.575	−0.01	0.09	−0.10	0.921	0.01	0.03	0.29	0.774
*F. hepatica* ++	0.10	0.03	3.17	**0.002**	-	-	-	-	−0.05	0.03	−1.71	0.089
*F. hepatica* +++	2.79 × 10^−3^	0.03	0.10	0.921	0.01	0.07	0.19	0.854	−0.05	0.04	−1.30	0.196
*D. viviparus* +	−2.29 × 10^−3^	0.03	−0.07	0.943	0.06	0.04	1.29	0.199	-	-	-	-
Breed type (DP vs. HD)	0.02	0.06	0.36	0.718	3.98	1.57	2.54	**0.012**	−0.66	0.17	−3.91	**<0.001**
Breed type (Mix vs. HD)	−0.11	0.06	−1.78	0.077	−0.26	0.13	−2.00	**0.047**	−0.66	0.20	−3.25	**0.001**
Lactation no.	−0.12	0.10	−1.24	0.217	−0.21	0.16	−1.28	0.202	−0.11	0.11	−1.02	0.308
Lactation no. (squared)	0.02	0.02	1.08	0.281	0.04	0.03	1.22	0.223	0.01	0.02	0.73	0.464
SCC (×1000/mL)	1.30 × 10^−4^	7.96 × 10^−5^	1.63	0.105	6.55 × 10^−5^	7.78 × 10^−5^	0.84	0.401	3.91 × 10^−4^	9.78 × 10^−5^	3.99	**<0.001**
Farm type (ORG vs. CON)	−0.10	0.04	−2.45	**0.015**	−0.10	0.03	−3.15	**0.002**	−0.10	0.03	−2.99	**0.003**
Farm type (TRA vs. CON)	-	-	-	-	-	-	-	-	−0.08	0.04	−1.75	0.082
Milk yield (kg)	-	-	-	-	−1.00 × 10^−5^	6.12 × 10^−6^	−1.64	0.104	4.40 × 10^−5^	1.92 × 10^−5^	−2.30	**0.023**
Breed type (DP)/Milk yield	-	-	-	-	−4.52 × 10^−4^	1.86 × 10^−4^	−2.43	0.016	7.52 × 10^−5^	2.02 × 10^−5^	3.72	**<0.001**
Breed type (Mix)/Milk yield	-	-	-	-	3.04 × 10^−5^	1.69 × 10^−5^	1.80	0.074	7.05 × 10^−5^	2.59 × 10^−5^	2.72	**0.007**

^a^ One outlier was excluded. Abbreviations: CON, conventional; Est., estimate; DP, dual-purpose; HD, high-performance dairy; ORG, organic; SCC, somatic cell count; SE, standard error; TRA, in transition from conventional to organic.

**Table 8:** Results from linear models investigating the association of *O. ostertagi*, *F. hepatica* and *D. viviparus* BTM ELISA results and average milk fat content (%) in three regions of Germany. Full models were significantly different from null models containing only an intercept term (North: Df = 14, F = 6.1, *p* < 0.001; East: Df = 13, F = 7.4, *p* = < 0.001; South: Df = 16, F = 2.7, *p* < 0.001). Significant *p*-values are printed in bold. Seropositivity categories are defined as follows: *O. ostertagi*: +: ODR ≥ 0.5, ++: ODR ≥ 0.8; *F. hepatica*: +: 30% < S/P ≤ 80%, ++: 80% < S/P < 150%, +++: S/P ≥ 150%; *D. viviparus*: +: ODR ≥ 0.41.

	North (N = 192)				East (N = 195) ^a^				South (N = 208)			
Variable	Est.	SE	*t*	*P*	Est.	SE	*t*	*p*	Est.	SE	*t*	*p*
Intercept	5.36	0.31	17.07	**<0.001**	5.26	0.45	11.67	**<0.001**	4.60	0.43	10.67	**<0.001**
*O. ostertagi* +	−0.03	0.03	−1.00	0.320	0.01	0.03	0.40	0.686	−0.04	0.04	−1.22	0.223
*O. ostertagi* ++	−0.04	0.05	−0.88	0.378	0.03	0.06	0.61	0.545	−0.13	0.06	−2.01	**0.046**
*F. hepatica* +	−0.02	0.06	−0.40	0.686	−0.08	0.19	−0.43	0.667	0.01	0.06	0.20	0.844
*F. hepatica* ++	0.11	0.07	1.60	0.111	-	-	-	-	−0.09	0.06	−1.68	0.094
*F. hepatica* +++	0.05	0.06	0.85	0.394	−0.11	0.14	−0.83	0.406	−0.12	0.06	−1.84	0.067
*D. viviparus* +	−0.01	0.07	−0.11	0.915	0.11	0.09	1.19	0.235	-	-	-	-
Breed type (DP vs. HD)	−0.12	0.13	−0.88	0.378	0.17	0.14	1.25	0.214	−0.49	0.29	−1.68	0.095
Breed type (Mix vs. HD)	−0.15	0.13	−1.16	0.249	0.01	0.06	0.15	0.885	−0.83	0.35	−2.35	**0.020**
Lactation no.	−0.34	0.21	−1.62	0.106	−0.35	0.33	−1.05	0.294	0.20	0.19	1.04	0.301
Lactation no. (squared)	0.06	0.04	1.77	0.079	0.07	0.06	1.19	0.236	−0.04	0.03	−1.29	0.200
SCC (×1000/mL)	−2.12 × 10^−4^	1.71 × 10^−4^	−1.24	0.216	−2.29 × 10^−4^	1.59 × 10^−4^	−1.45	0.150	2.36 × 10^−4^	1.68 × 10^−4^	1.41	0.161
Herd size	−4.87 × 10^−4^	2.67 × 10^−4^	−1.83	0.069	1.21 × 10^−5^	4.14 × 10^−5^	0.29	0.770	−3.88 × 10^−4^	3.28 × 10^−4^	−1.18	0.238
farm type (ORG vs. CON)	−0.16	0.08	−1.89	0.061	−0.09	0.07	−1.34	0.183	−0.06	0.06	−1.07	0.287
farm type (TRA vs. CON)					-	-	-	-	−0.06	0.08	−0.72	0.472
Milk yield (kg)	−7.70 × 10^−5^	1.15 × 10^−5^	−6.69	**<0.001**	−8.12 × 10^−5^	1.27 × 10^−5^	−6.42	**<0.001**	−7.58 × 10^−5^	3.37 × 10^−5^	−2.25	**0.025**
Breed type (DP)/Milk yield	-	-	-	-	-	-	-	-	5.16 × 10^−5^	3.51 × 10^−5^	1.47	0.143
Breed type (Mix)/Milk yield	-	-	-	-	-	-	-	-	1.04 × 10^−4^	4.50 × 10^−5^	2.31	**0.022**

^a^ One outlier was excluded. Abbreviations: CON, conventional; Est., estimate; DP, dual-purpose; HD, high-performance dairy; ORG, organic; SCC, somatic cell count; SE, standard error; TRA, in transition from conventional to organic.

## Data Availability

Data supporting reported results is contained within the article and the Supplementary Materials.

## Supplementary Materials

The following are available online at https://www.mdpi.com/article/10.3390/ani11072078/s1, Table S1: Qualitative predictor variables for logistic regression regarding management factors associated with *O. ostertagi*, *F. hepatica* and *D. viviparus* BTM ELISA results; Table S2: Results from linear models investigating the association of *O. ostertagi* and *F. hepatica* BTM ELISA results with average annual milk yield (kg), average milk protein (%) and average milk fat (%) content in high performance dairy (HD) and dual-purpose (DP) herds in southern Germany.
