# Supplementary material for: Seroprevalence of Major Pasture-Borne Parasitoses (Gastrointestinal Nematodes, Liver Flukes and Lungworms) in German Dairy Cattle Herds, Association with Management Factors and Impact on Production Parameters
Source: Animals (Basel). 2021 Jul 12;11(7):2078. doi: 10.3390/ani11072078 (PMC8300236; doi:10.3390/ani11072078)
Supplement: Supplementary file 1 [file animals-11-02078-s001.zip › Table S1_management descriptive.pdf]

Table S1. Qualitative predictor variables for logistic regression regarding management factors associated with *O. ostertagi*, *F. hepatica* and *D. viviparus* BTM ELISA results. Abbreviations: N, Number of farms

	Northwest		Northeast		Bavaria		Total	
	**N**	**%**	**N**	**%**	**N**	**%**	**N**	**%**
**Access to pasture/fresh grass**								
Yes	96	47.76	51	24.88	77	31.95	224	34.62
No	101	50.25	142	69.27	146	60.58	389	60.12
Unknown	4	1.99	12	5.85	18	7.47	34	5.26
**Floor dried hay**								
Floor dried hay [a]	2	1.00	14	6.83	47	19.50	63	9.74
Other dried hay	0	0	0	0	8	3.32	8	1.24
No or low hay percentage in ration	196	97.51	189	92.20	182	75.52	567	87.64
Unknown	3	1.49	2	0.98	4	1.66	9	1.39
**Silage and hay quality**								
Normal/slightly lower quality	80	39.80	121	59.02	112	46.47	313	48.38
At least 1 silage with significantly lower quality	14	6.97	14	6.83	25	10.37	53	8.19
At least 1 spoiled silage	102	50.75	61	29.76	93	38.59	256	39.57
Unknown	5	2.49	9	4.39	11	4.56	25	3.86
**Presence of fully slatted floor**								
Yes [b]	1	0.50	1	0.49	6	2.49	8	1.24
No	200	99.50	203	99.02	235	97.51	638	98.61
Unknown	0	0	1	0.49	0	0	1	0.15
**Anthelmintic treatment in calves[c] and young cattle[d]**								
Calves and young cattle	34	16.92	7	3.41	18	7.47	59	9.12
Only calves [e]	3	1.49	4	1.95	3	1.24	10	1.55
Only young cattle	111	55.22	68	33.17	67	27.80	246	38.02
No anthelmintic treatment	50	24.88	125	60.98	152	63.07	327	50.54
Unknown	3	1.49	1	0.49	1	0.41	5	0.77
**Anthelmintic treatment in lactating and dry cows**								
Lactating and dry cows	81	40.30	27	13.17	36	14.94	144	22.26
Only dry cows	7	3.48	12	5.85	7	2.90	26	4.02
Only lactating cows[e]	14	6.97	9	4.39	4	1.66	27	4.17
No anthelmintic treatment	99	49.25	157	76.59	194	80.50	450	69.55
**All**	201	100.00	205	100.00	241	100.00	647	100.00

[a] If < 5 farms in the category, these were counted as "no or low hay percentage in ration"
[b] If < 5 farms in the category, these were counted as "no presence of fully slatted floors"
[c] Pre-weaning or < 6 months of age
[d] Weaning/6 months of age to first calving
[e] If < 5 farms in the category, these were counted as "no anthelmintic usage"
