# Supplementary material for: Seroprevalence of Major Pasture-Borne Parasitoses (Gastrointestinal Nematodes, Liver Flukes and Lungworms) in German Dairy Cattle Herds, Association with Management Factors and Impact on Production Parameters
Source: Animals (Basel). 2021 Jul 12;11(7):2078. doi: 10.3390/ani11072078 (PMC8300236; doi:10.3390/ani11072078)
Supplement: Supplementary file 1 [file animals-11-02078-s001.zip › Table S2_Breeds.pdf]

Table S2: Results from linear models investigating the association of *Ostertagia ostertagi* and *Fasciola hepatica* BTM ELISA results with average annual milk yield (kg), average milk protein (%) and average milk fat (%) content in high performance dairy (HD) and dual-purpose (DP) herds in southern Germany. Seropositivity categories are defined as follows: *O. ostertagi*: +: ODR ≥ 0.5, ++: ODR ≥ 0.8; *F. hepatica*: +: 30% < S/P ≤ 80%,++: 80% < S/P < 150%, +++: S/P ≥ 150%.

	HD (N = 27)[a]				DP (N = 161)			
	**Estimate**	**SE**	**t**	***P*-value**	**Estimate**	**SE**	**t**	***P*-value**
**Milk yield (kg)[b]**								
Intercept	14852.65	5802.40	2.56	**0.020**	8951.27	1746.67	5.13	**< 0.001**
O. ostertagi +	117.36	330.26	0.36	0.727	-103.93	248.01	-0.42	0.676
O. ostertagi ++	-	-	-	-	-107.09	406.23	-0.26	0.792
F. hepatica +	-939.71	457.04	-2.06	0.056	19.18	499.30	0.04	0.969
F. hepatica ++	-296.89	578.31	-0.51	0.614	-363.50	347.90	-1.05	0.298
F. hepatica +++	-2411.40	898.22	-2.69	**0.016**	843.24	384.11	2.20	**0.030**
Lactation no.	-3882.50	4028.61	-0.96	0.349	-668.34	1134.60	-0.59	0.557
Lactation no. (squared)	586.24	662.85	0.88	0.389	104.52	183.55	0.57	0.570
SCS	-1.50	2.33	-0.65	0.528	-3.12	0.95	-3.27	**0.001**
No. of lactating animals	7.83	3.27	2.39	**0.029**	9.97	3.49	2.86	**0.005**
Farm type (ORG vs. CON)	271.69	644.91	0.42	0.679	-1606.45	340.18	-4.72	**< 0.001**
Farm type (TRA vs. CON)	-	-	-	-	-523.23	452.80	-1.16	0.250
**Protein content (%)[c]**								
Intercept	4.64	1.11	4.17	**0.001**	3.61	0.20	18.41	**< 0.001**
O. ostertagi +	0.07	0.06	1.12	0.278	0.01	0.03	0.46	0.647
O. ostertagi ++	-	-	-	-	0.01	0.04	0.31	0.756
F. hepatica +	-0.06	0.10	-0.63	0.535	-0.04	0.05	-0.82	0.414
F. hepatica ++	0.05	0.11	0.43	0.671	-0.07	0.04	-1.94	0.054
F. hepatica +++	-0.43	0.20	-2.18	**0.043**	-0.04	0.04	-0.96	0.339
Lactation no.	-0.42	0.71	-0.59	0.563	-0.22	0.12	-1.87	0.063
Lactation no. (squared)	0.08	0.12	0.66	0.520	0.03	0.02	1.57	0.119
SCS	9.24×10^{-5}	4.45×10^{-4}	0.21	0.838	3.71×10^{-4}	1.04×10^{-4}	3.58	**< 0.001**
farm type (ORG vs. CON)	0.08	0.12	0.64	0.533	-0.11	0.04	-2.93	**0.004**

farm type (TRA vs. CON)	-	-	-	**-**	-0.09	0.05	-1.81	0.073
Milk yield	-6.28×10^{-5}	3.96×10^{-5}	-1.59	0.131	2.82×10^{-5}	8.37×10^{-5}	3.36	**0.001**
**Milk fat (%)**[d]								
Intercept	2.74	1.63	1.69	0.111	4.15	0.35	11.83	**<0.001**
O. ostertagi +	-0.13	0.08	-1.58	0.133	-0.07	0.05	-1.59	0.114
O. ostertagi ++	-	-	-	-	-0.15	0.08	-2.00	**0.047**
F. hepatica +	-0.10	0.12	-0.85	0.408	0.06	0.09	0.61	0.545
F. hepatica ++	-0.13	0.14	-0.93	0.365	-0.07	0.06	-1.08	0.280
F. hepatica +++	-0.05	0.26	-0.20	0.845	-0.06	0.07	-0.89	0.377
Lactation no.	1.80	0.99	1.83	0.086	0.15	0.21	0.73	0.467
Lactation no. (squared)	-0.32	0.16	-1.97	0.066	-0.03	0.03	-0.92	0.357
SCS	-1.18×10^{-3}	5.62×10^{-4}	-2.10	0.052	3.36×10^{-4}	1.83×10^{-4}	1.83	0.069
No. of producing animals	-1.68×10^{-3}	9.01×10^{-4}	-1.87	0.080	-1.38×10^{-4}	6.64×10^{-4}	-0.21	0.836
Farm type (ORG vs. CON)	0.01	0.15	0.05	0.961	-0.07	0.07	-1.00	0.318
Farm type (TRA vs. CON)	-	-	-	-	-0.07	0.08	-0.84	0.401
Milk yield	-6.93×10^{-5}	5.78×10^{-5}	-1.20	0.248	-2.54×10^{-5}	1.52×10^{-5}	-1.67	0.097

[a] One outlier was excluded

[b] Full models were significantly different from null models containing only an intercept (HD: Df = 9, F = 6.9, $P < 0.001$; DP: Df = 11, F = 6.8, $P < 0.001$)

[c] Only the DP model was significantly different from a null model containing only the intercept (HD: Df = 9, F = 0.99, $P = 0.480$; DP: Df = 11, F = 6.2, $P < 0.001$)

[d] Only the DP model was significantly different from a null model containing only the intercept (HD: Df = 10, F = 2.2, $P = 0.079$; DP: Df = 12, F = 2.7, $P = 0.003$)

Abbreviations: ORG, organic; CON, conventional; TRA, in transition from conventional to organic; SE, standard error; SCS, somatic cell score
